# High Prevalence of *Candida* Yeast in Milk Samples from Cows Suffering from Mastitis in Poland

**DOI:** 10.1100/2012/196347

**Published:** 2012-04-24

**Authors:** Bozena Dworecka-Kaszak, Alicja Krutkiewicz, Daniel Szopa, Miroslaw Kleczkowski, Malgorzata Biegańska

**Affiliations:** ^1^Mycology Division, Preclinical Science Department, Faculty of Veterinary Medicine, Warsaw University of Life Sciences, Ciszewskiego 8, 02-789 Warsaw, Poland; ^2^Molecular Genetic Division, Mother and Child institute, Kasprzaka 17a, 01-211 Warsaw, Poland; ^3^Division of Laboratory Diagnostics, Department of Pathology and Veterinary Diagnostics, Faculty of Veterinary Medicine, Warsaw University of Life Sciences, Nowoursynowska 159, 02-776 Warsaw, Poland

## Abstract

Mastitis is an economically important disease in which fungi belonging to the genus *Candida* may participate as etiological agents. This study focused on determining the frequency of fungal isolation and differentiation of fungal species isolated from milk of mastitic cows. Sixty-six milk samples from mastitic cows were studied, and 55 strains of fungi were isolated. Seven different species classified as *Candida* were identified basing on phenotypic properties, and the dominating species was *C. parapsilosis*. Genomic DNA was isolated and amplified in PCR with ITS1 and NL2 primers. Amplification products were digested with restriction enzymes *Hpa*II and *EcoR*I. Amplification of DNA with ITS1 and NL2 primers resulted in products of different sizes. Comparison of product sizes in restriction fragment PCR REA confirmed differences among species. Strains grouped together on the basis of phenotype characteristics differed in restriction fragment profiles. None of the investigated species showed similar genetic profiles.

## 1. Introduction

Mastitis in dairy cattle is an inflammatory reaction of the udder. Infection of the mammary gland is the most common and most costly disease in the dairy industry. The symptoms of mastitis include abnormalities such as a watery appearance of milk, flakes, clots, or pus in milk [1–3]. Mastitis leads to a decline in potassium, lactoferrin, and casein content in milk. Because calcium in milk is associated with casein, the disruption of casein synthesis contributes to lower levels of calcium in milk. Milk from cows with mastitis also has a higher somatic cell count which lowers the quality of milk. Mastitis occurs when leucocytes are released into the mammary gland, usually in response to invasion by microorganisms through the teat canal. This disease can be identified by external symptoms such as swelling, heat, redness, hardness, or pain of the udder. There are many bacteria known to cause mastitis which include *Pseudomonas aeruginosa, Staphylococcus aureus, Staphylococcus epidermidis*, *Streptococcus agalactiae, Brucella melitensis*, *Corynebacterium bovis, Mycoplasma sp., Escherichia coli, Klebsiella pneumoniae, Klebsiella oxytoca, Enterobacter aerogenes, Pasteurella sp., Arcanobacterium pyogenes, Proteus sp., *and* Prototheca sp.* (algae) [1–3]. Several species of yeast or yeast-like microorganisms have been reported to cause bovine mastitis [4, 5]. *Cryptococcus neoformans* and *Candida albicans* are by far the most common causes, but other *Candida* species have also been associated with bovine mastitis. Mastitis is usually transmitted through contaminated milking machines and milker's hands or other materials. Treatment is possible with long-acting antibiotics, but milk from such cows is not marketable until drug residues have left the cow's system. Antibiotics may be administered systemically, or they may be applied locally by upward force through the teat canal. Antibiotic therapy, without identifying the mastitis-causing organisms, is frequently the veterinarian and dairy farmer's first choice of treatment for diseased cows. As a result, cases of mastitis (including fungal mastitis) that are refractory to any type of treatment occur frequently.

The incidence of mastitis due to yeast is usually low in dairy herds, but it has significantly increased during the last decade. Fungal mastitis has been described as related to treatment directed against other pathogens using contaminated syringes, canulars, or contaminated antibiotic preparations. Teat injuries may predispose to establishment of a yeast infection. Yeast intramammary infections have been reported to be responsible for at least 10% of all clinical cases seen in veterinary practice [1–3], and the majority of the cases are usually mild. Although antimycotic drugs have been used for treatment of yeast mastitis, there is no clear evidence of the effectiveness of this therapy [1–5].

The aim of the present study was to isolate, identify, and determine the prevalence of yeast in milk samples from cows suffering from mastitis in Poland.

## 2. Materials and Methods

Sixty-six quarter-milk samples were collected from 44 cows with clinical or subclinical mastitis on small farms in the North of Poland (Łomża) or near Warsaw. Samples were plated onto Blood Agar, Macconkey Agar, and Sabouraud Dextrose Agar and incubated at 37°C for 72 h for bacteria growth and for 2 weeks for yeast. Fungi were identified phenotypically, and selected species were also identified genotypically. The genera and species of yeast were identified by API *Candida* (API C), API ID32C tests (bioMerieux), and germ tube test. Bacteria were identified and classified according to Bergey's Manual of Systematic Bacteriology [6], using morphological and biochemical characteristics and by API strip tests (API20E, API Staph, API Coryne, and API Strept).

### 2.1. Isolation and Amplification of *Candida* DNA

A PCR-based method was used to verify the identity of 20 *Candida* strains. Genomic DNA was isolated with Genomic Mini-AX Yeast Kit (DNA-Gdańsk) according to the manufacturer's protocol. DNA isolation was verified by separation on 0.8% agarose gel with ethidium bromide in TBE buffer. Electrophoresis was performed for 1 h at 100 V, and the gels were analysed in VersaDoc (BioRad) gel documentation system and quantified by Quantity One software (BioRad). To further identify the species, a 629 bp DNA fragment containing the gene encoding 5,8S rRNA and ITS sequence was amplified with 2 primers as described by Skała et al. [7], with a slight modification

ITS1 5′-TCCGTAGGTGAACCTGCGG-3′

NL2 5′-CTCTCTTTTCAAGTGCTTTTCATCT-3′.

Primers were produced by DNA IBB, PAN, Poland. The reaction mixture (50 *μ*L) contained 50 ng yeast genomic DNA, 1 U *Taq* polymerase (Fermentas), buffer for *Taq* polymerase (Fermentas), 2 mM of each dNTP (Fermentas), 20 mM MgCl_2,_ and 1 mM each primer. Amplification was performed in Mastercycler thermal cycler according to the following protocol: denaturation at 95°C for 5 min., then 25 cycles containing denaturation at 95°C for 45 sec., annealing at 50°C for 45 sec., first extention/elongation at 72°C for 90 sec., and final elongation at 72°C for 15 min. To separate amplicons, gel electrophoresis was performed (1 h/80 V). Gels were read in VersaDoc (BioRad) by Quantity One program. 

### 2.2. Restriction Endonuclease Analysis

To compare *Candida* isolates within the genus, amplification products were digested by 2 restriction endonucleases: *Hpa*II and *EcoR*I (Fermentas) [8, 9]. For this purpose, 10 *μ*L of the PCR product, 2 *μ*L of restriction enzyme, and 6 *μ*L of buffer were mixed and incubated for 2 h at 37°C and then heated at 65°C for 20 min to inactivate the enzymes. Restriction fragments were resolved by agarose gel electrophoresis (2 h/90 V) and analyzed by Quantity One software.

## 3. Results

**Figure 1:**
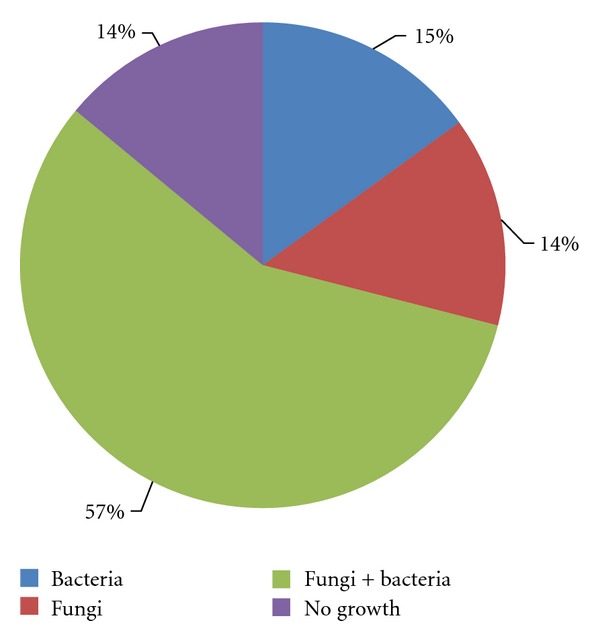
Cultivation results of milk samples from cows suffering from mastitis.

**Figure 2:**
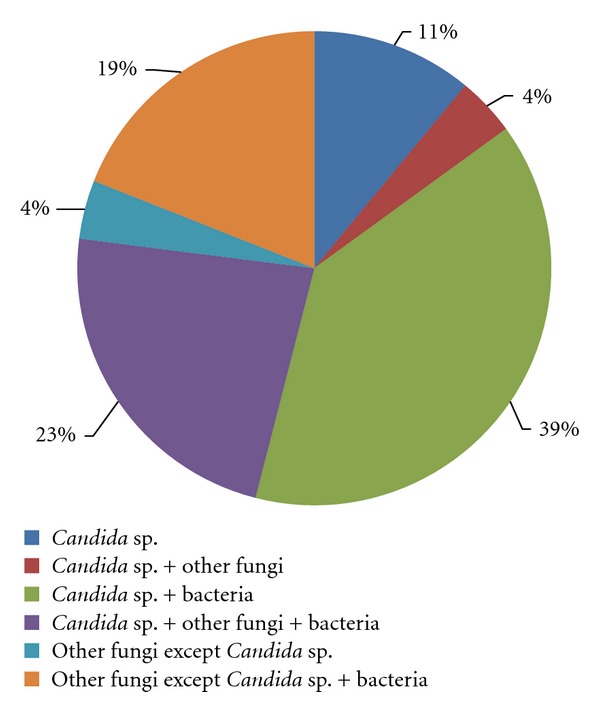
Percentage distribution of different mastitis aetiological agents isolated from milk samples.

**Figure 3:**
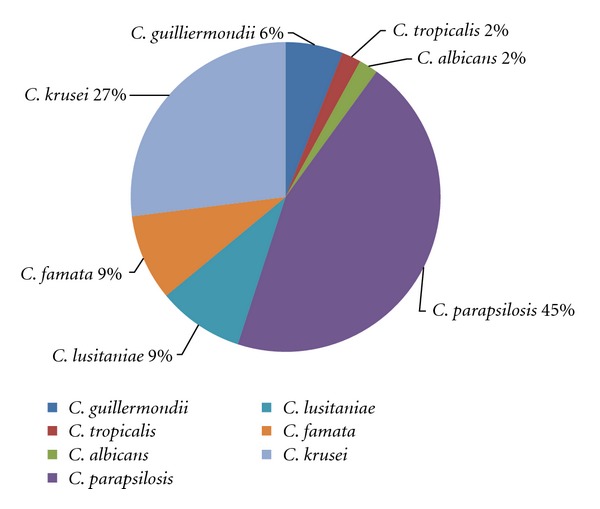
Proportion of* Candida *species isolated from milk samples of cows suffering from mastitis.

Fifty-five* isolates belonging to Candida *genus were cultured from 66 milk samples examined (Figures 1 and 2). 25 were *Candida parapsilosis, *15 were *Candida krusei, *5 were *Candida lusitaniae, *5 were *Candida famata, *3 were *Candida guilliermondii, *1 was *Candida tropicalis, *and 1 was *Candida albicans *(Figure 3). Additionally, 27 other fungi were isolated, among which 9 were *Cryptococcus *genus, 7 were *Trichosporon* genus, 5 were isolates of *Saccharomyces* genus, 3 were isolates of *Geotrichum* genus, and 3 were *Rhodotorula *genus. Growth of mycelial fungi was treated as contamination. Bacteria typical for mastitis were also isolated. Results are shown in Table 1. 

Genomic DNA was isolated from 20 randomly chosen fungal isolates identified as members of *Candida* genus and amplified with ITS1 and NL2 primers. The investigated isolates were classified into seven different species (on the base of their phenotypic properties with assistance of striped test evaluated API *Candida* and API ID 32C bioMerieux): 3 isolates of* C. krusei, *3 isolates of* C. parapsilosis, *5 isolates of *C. famata,* 3 isolates of *C. guilliermondii, *4 isolates of *C. lusitaniae, *1 of *C. albicans, *and 1 isolate of *C. tropicalis.* Amplification of DNA fragments with ITS1 and NL2 primers revealed products of different sizes, ranging from 700 to 1000 bp dependent on species from which genetic material was isolated (Table 2).

PCR amplification products were digested by restriction enzymes *Hpa*II and *EcoR*I (Table 3). Comparison of restriction fragments confirmed differences among species (Table 3). However, isolates qualified on the base of phenotype into one species differed greatly in restriction fragment profiles. None of the investigated species revealed a characteristically similar genetic profile. Results reported here indicate that isolates of fungi phenotypically similar, such as *Candida* species, may exhibit a differentiated genotype regarding location of restriction sites of the same restriction enzyme.

## 4. Discussion

The fungi of *Candida* sp. generally grow well on Sabouraud Agar at 37°C, usually forming colonies within 24–48 h. Colonies are opaque, often white or yellowish, and at first usually smooth. Their texture is creamy or pasty, and in a microscopic smear it appears to consist solely of oval to round budding blastospores. In our examination, we noted that yeast from milk of mastitic cows required more time to form colonies during cultivation. Most colonies appeared at 48–72 h after the milk samples were plated, but some strains needed almost one week to grow on the medium. In our opinion, the delayed growth could have been a result of abnormal conditions previously found in the milk (acidic pH, lack of proteins, etc.) such that the fungi needed more time to adapt to growth in a new environment. This observation suggests that fungal growth may be underestimated if growth observation is terminated early.

 Our investigation shows a high frequency of bacterial-fungal infections, approximately 57% of all examined samples. Bacteria or fungi as sole etiological agents were found in 15% and 14%, respectively, of investigated samples. *Candida* yeast was isolated from 39% of the mixed infections, and in 11% of these cases, it was a sole infectious agent.

Krukowski et al. [10] isolated fungi as pure cultures from 9.6% of investigated milk samples in the Lublin region in Poland, a result much similar to ours. According to Casia dos Santos and Mo acir Marin [11], the percentage of fungal isolation in surveys carried out in many countries varies considerably, with 6,1% rates described in Egypt [12], 1,3% in Denmark [13], and 12,07% in Brazil [14]. Casia dos Santos and Moacir Marin [11] isolated fungi in 32% of the cases, and 17.3% of these were *Candida* spp.

*Candida* is commonly viewed as an opportunistic yeast pathogen, and the source of infection may be skin of the udder, milker's hands, milking machines, floors, straw, feed, medications, sanitary agents, and other equipment [15, 16]. Under immunosuppressive conditions, the population size balance may be disrupted, and the fungi together with the other microorganisms are able to overcome the udder defense mechanisms. Although the distribution of *Candida *species shows diversity in several countries, it is important to note the increase in number of mammary gland infections caused by *Candida *species in the recent years. Krukowski et al. [10] reported that the most frequently isolated species in Poland were *C. kefyr, C. cirferi,* and *C. krusei. * In the other reports, more frequent were *C. krusei, C. rugosa, *and *C. albicans, *as described by Costa et al. [2, 14, 16] or Şeker [15]. In our investigation, the most frequently isolated species was *C. parapsilosis* (25 strains) followed by *C. krusei* (15 strains). *Candida albicans* was isolated only from one sample (confirmed by positive germ tube test). In our experiments, *Candida* strains were first identified phenotypically by API *Candida* (API C) and API ID 32C tests (bioMerieux) and germ tube test and then confirmed by genotypic methods. Comparison of PCR product size confirmed differences between species. However, isolates qualified into one species on the basis of phenotypic characteristics alone differed genotypically. We did not generate comparable genetic profiles in any of the species investigated, particularly in the strains originating from the North of Poland and the Warsaw area. Our results indicate that strains of fungi phenotypically classified as the same *Candida* species can have different genotypes generated by restriction endonuclease analysis. This high interspecies heterogeneity may suggest large environmental adaptive properties of *Candida* strains.

## Figures and Tables

**Table 1:** Bacteria isolated from milk samples of mastitic cows.

Species	Number of isolates
*Streptococcus agalactiae*	5
*Streptococcus α* *-hemolytic *	7
*Staphylococcus coagulase-*negative	3
*Staphylococcus aureus*	6
*Escherichia coli*	15
*Proteus *sp.	8
*Corynebacterium* sp.	2

Total	46

**Table 2:** PCR products of *Candida* of genomic DNA generated with the ITS1 and NL2 primers. Black bold type fonts shows Warsaw isolates.

Species	Strains	Product size [bp]
*C. famata*	1	950
	2	950
	3	750
	4	750
	5	**1050**

*C. krusei*	1	700
	2	650
	3	650

*C. parapsilosis*	1	950
	2	840
	3	700

*C. guilliermondii*	1	**800**
	2	700
	3	700

*C. lusitaniae*	1	800
	2	850
	3	1000
	4	1000

*C. tropicalis*	1	1000

*C. albicans*	1	900

**Table 3:** Restriction endonuclease analysis of PCR products digested with *Hpa*II and *EcoR*I. Black bold type fonts shows Warsaw isolates.

Size of products after digestion with restriction enzymes [bp]		Strains	Species
*Hpa*II	*EcoR*I		
950	950	1	*C. famata*
950	950	2	
420 330	750	3	
420 330	750	4	
**600 450**	**>1000**	5	

**410 400 **	**800 **	1	*C. guilliermondii*
400 300	600 100	2	
400 300	600 100	3	

1000	1000	1	*C. tropicalis*

450 450	900	1	*C. albicans*

700	700	1	*C. krusei*
400 250	650	2	
400 250	650	3	

330	800	1	*C. lusitaniae*
350 300 200	850	2	
1000	1000	3	
1000	1000	4	

950	950	1	*C. parapsilosis*
350 250 240	840	2	
400 300	700	3	

## References

[1] Krukowski H (2001). Mycotic mastitis in cows. *Medycyna Weterynaryjna*.

[2] Costa EO, Ribeiro AR, Watanabe ET, Melville PA (1998). Infectious bovine mastitis caused by environmental organisms. *Journal of Veterinary Medicine Series B*.

[3] Jones GM Understanding the Basics of Mastitis. http://pubs.ext.vt.edu/404/404-233/404-233.html.

[4] Staroniewicz Z, Włodarczak A, Florek M, Król J (2007). Fungal flora in cows with mastitis and its susceptibility to antimycotics. *Mikologia Lekarska*.

[5] Sheena A, Sigler L (1995). *Candida krusei* isolated from a sporadic case of bovine mastitis. *Canadian Veterinary Journal*.

[6] (1994). *Bergey‘s Manual of Determinative Bacteriology*.

[7] Skała J, Potocka N, Bortniczuk M Molecular identification strains from *Candida, Cryptococcus, Hansenula, Rhodotorula* and *Trichosporon* genus.

[8] Campos De Pinho Resende J, Franco GR, Rosa CA, Hahn RC, Soares Hamdan J (2004). Phenotypic and genotypic identification of *Candida* spp. isolated from hospitalized patients. *Revista Iberoamericana de Micologia*.

[9] Mecler I, Nawrot U (2008). Molecular methods of *Candida* identifiction. *Mikologia Lekarska*.

[10] Krukowski H, Tietze M, Majewski T, Rózański P (2001). Survey of yeast mastitis in dairy herds of small-type farms in the Lublin region, Poland. *Mycopathologia*.

[11] De Casia Dos Santos R, Marin JM (2005). Isolation of *Candida* spp. from mastitic bovine milk in Brazil. *Mycopathologia*.

[12] Awad FI, El Molla A, Fayed A, Abd el-Halim M, Refai M (1980). Studies of mycotic mastitis in Egypt. *Journal of Egyptian Veterinary Medical Association*.

[13] Aalbek B, Stenderup J, Jensen HE, Valbak J, Nylin B, Huda A (1994). Mycotic and algal bovine mastitis in Denmark. *Acta Pathologica, Microbiologica et Immunologica*.

[14] Costa EO, Gandra CR, Pires MF, Coutinho SD, Castilho W, Teixeira CM (1993). Survey of bovine mycotic mastitis in dairy herds in the State of São Paulo, Brazil. *Mycopathologia*.

[15] Şeker E (2010). Identification of *Candida* species isolated from bovine mastitic milk and their in vitro hemolytic activity in western Turkey. *Mycopathologia*.

[16] Da Costa EO, Ribeiro M, Ribeiro AR, Rocha NS, Júnior GDN (2004). Diagnosis of clinical bovine mastitis by fine needle aspiration followed by staining and scanning electron microscopy in a Prototheca zopfii outbreak. *Mycopathologia*.

